# Association of Suppressive Myeloid Cell Enrichment with Aggressive Oropharynx Squamous Cell Carcinoma

**DOI:** 10.3390/cancers15082346

**Published:** 2023-04-18

**Authors:** Changlin Yang, Rekha Garg, Kristanna Fredenburg, Frances Weidert, Hector Mendez-Gomez, Robert Amdur, Ji-Hyun Lee, Jamie Ku, Jesse Kresak, Stephanie Staras, Andrew G. Sikora, Lily Wang, Daniel McGrail, Duane Mitchell, Elias Sayour, Natalie Silver

**Affiliations:** 1 Department of Neurosurgery, University of Florida, Gainesville, FL 32608, USA; 2 Department of Pediatrics, University of Florida, Gainesville, FL 32603, USA; 3 Department of Pathology, University of Florida, Gainesville, FL 32610, USA; 4 Department of Radiation Oncology, University of Florida, Gainesville, FL 32608, USA; 5 Department of Biostatistics, University of Florida, Gainesville, FL 32603, USA; 6 Head and Neck Institute, Cleveland Clinic, Cleveland, OH 44106, USA; 7 Department of Health Outcomes and Biomedical Informatics, University of Florida, Gainesville, FL 32610, USA; 8 Department of Head and Neck Surgery, MD Anderson Cancer Center, University of Texas, Houston, TX 77030, USA; 9 Translational Hematology and Oncology, Cleveland Clinic, Cleveland, OH 44195, USA; 10 Lerner Research Institute, Cleveland Clinic, Center of Immunotherapy and Precision Immuno-Oncology, Cleveland, OH 44106, USA

**Keywords:** head and neck cancer, oropharynx cancer, human papillomavirus, myeloid cell, TCGA

## Abstract

**Simple Summary:**

Human papillomavirus positive (HPV+) ororpharyngeal squamous cell carcinoma (OPSCC) patients have improved clinical prognosis, compared to HPV-negative OPSCC patients. While HPV+ OPSCC patients often have more immune-cell-infiltrated tumors, they can be subjected to increased immunoregulatory influence. The purpose of this study was to examine the tumor immune microenvironment, via mRNA expression profiling, in patients with OPSCC, focusing primarily on HPV+ patients with aggressive disease (patients with known recurrent or metastatic disease). Using primary-patient pre-treatment tumor tissue samples, we demonstrated that HPV-negative and aggressive-HPV+ OPSCC patients have increased monocyte/macrophage and granulocyte progenitor cell enrichment. We examined The Cancer Genome Atlas (TCGA) database for head and neck cancer to find similar trends in myeloid cell enrichment in more aggressive OPSCC.

**Abstract:**

Background: While immune-cell infiltrated tumors, such as human papillomavirus positive (HPV+) ororpharyngeal squamous cell carcinomas (OPSCC) have been associated with an improved clinical prognosis, there is evidence to suggest that OPSCCs are also subjected to increased immunoregulatory influence. The objective of this study was to assess whether patients with clinically aggressive OPSCC have a distinct immunosuppressive immune signature in the primary tumor. Methods: This retrospective case-control study analyzed 37 pre-treatment tissue samples from HPV+ and HPV-negative OPSCC patients treated at a single institution. The cases were patients with known disease recurrence and the controls were patients without disease recurrence. An mRNA-expression immune-pathway profiling was performed, and correlated to clinical outcomes. The TCGA head and neck cancer database was utilized to make comparisons with the institutional cohort. Results: In our cohort, HPV-negative and HPV+ patients with known disease recurrence both had significantly increased suppressive monoctyte/macrophage and granulocyte cell-expression-profile enrichment. Similar findings were found in the TCGA cohort when comparing HPV-negative to positive patients. Conclusions: our study demonstrates that patients with recurrent HPV+ OPSCC had suppressive monocyte/macrophage and granulocyte immune-cell enrichment, similar to those seen in the more aggressive HPV-negative OPSCC.

## 1. Introduction

The incidence of human papillomavirus (HPV)-associated oropharyngeal squamous cell carcinoma (OPSCC) has more than doubled from 1988 to 2004, and at the same time, the incidence of HPV-negative cancers has declined by more than 50% [1]. In the United States, the percentage of OPSCC that are HPV+ continues to increase, and accounts for 65% of all OPSCCs during 2000–2013 [2]. HPV status is an independent prognostic factor for survival among patients with OPSCC. The 3-year overall survival rates are 82% in HPV positive (HPV+) OPSCC patients and only 57% in HPV-negative cases, despite the fact that HPV+ patients usually have poorly differentiated histology, and often present with regionally metastatic disease [3]. Although patients with HPV-associated OPSCC have a very good prognosis, the tumors can be biologically diverse: in 20–25% of patients the symptoms will recur in the first 5 years, and a significant proportion of these patients will die from recurrent disease [3]. While heavy smoking has been associated with worse clinical outcomes for patients with HPV+ tumors, specific mechanisms of disease recurrence in this population still remain unclear [4,5].

Studies have investigated tumor-infiltrating immune cells in head and neck cancer, and demonstrated differences between HPV+ and HPV-negative tumor-infiltrating immune profiles. Specifically, over-representation of certain immune cell types has been linked to prognosis [6]. For example, in some studies on human tumor samples, tumor-infiltrating T lymphocytes such as CD8+ T cells have been associated with a favorable prognosis in HPV+ OPSCCs [7,8,9,10,11]. Macrophages (myeloid lineage cells), on the other hand, can be pro-inflammatory (M1-like) and immunosuppressive (M2-like), and can have opposing effects in the tumor microenvironment. Tumor-associated macrophages (TAMs) in head and neck cancer, have been associated with more-advanced-stage disease, increased tumor invasiveness, and increased tumor aggression, suggesting that pro-tumorigenic macrophages predominate in head and neck squamous cell carcinoma (HNSCC) [12,13]. In addition to macrophages, myeloid-derived suppressor cells (MDSCs) are innate immune cells that can suppress immune anti-tumoral responses and have been linked to worse prognoses when present in the tumor microenvironment in HNSCCs [14,15]. These suppressive immune cells are derived from both myeloid and granulocyte progenitor cells.

In this study, we used RNA expression profiling of pre-treatment surgical biopsies to examine the immunologic and genetic profiles for patients who have known recurrence (compared to patients without recurrence) following treatment for OPSCC. Additionally, we examined the immune profiles of the TCGA head-and-neck-cancer patient cohort to draw comparisons with our institutional cohort. 

## 2. Materials and Methods

### 2.1. Biospecimens

The Institutional Review Board (IRB) and office of human research ethics at the University of Florida approved this study. Patients with HPV+ and HPV-negative OPSCC were identified from the clinical database for who had been treated at the University of Florida between 2011 and 2019 (to ensure adequate follow-up time) and had pre-treatment biopsies with sufficient material available for analysis. Banked formalin-fixed paraffin-embedded (FFPE) tumor tissue was procured from 37 specimens from patients with a diagnosis of OPSCC. We focused our specimen selection to capture HPV+ patients with recurrence. Based on institutional numbers and survival rates, we expect approximately 2.4 patients per year to die of disease or recurrence (approximately 20 patients per year with HPV+ OPSCC are treated at the institution with ~89% 3-year overall survival in this population) [16]. We were able to identify 20 patients with HPV+ OPSCC recurrent disease, adequate follow-up, and tissue for analysis. This number accurately captures the patients from expected rates of failure in this patient population during this 8-year time period. A p16-positive status as a surrogate for HPV status was confirmed by immunohistochemistry (IHC), based on the review of patient pathology reports. Institutional protocols were followed, in which IHC staining for p16 is performed using the CINtec Histology Kit (Roche Laboratories), and samples with >70% strong diffuse nuclear and cytoplasmic staining of tumor cells are positive [17,18,19,20]. A total of n = 20 samples were from patients with recurrence and n = 17 patients without recurrence. Recurrence was defined as local and/or regional disease recurrence and/or distant failure. The four groups included for analysis were HPV+ patients with recurrence (HPV+R+), HPV+ patients without recurrence (HPV+R-), HPV-negative patients with recurrence (HPV-R+) and HPV-negative patients without recurrence (HPV-R-). Tumor blocks were sectioned into five 10-μm sections by the Tissue Pathology Core Facility at the University of Florida, and FFPE sections were then placed onto glass slides. For each tumor, 1 hematoxylin-eosin–stained slide was prepared prior to nucleic acid harvest, analyzed, and marked by a board-certified pathologist (JK) to identify the tumor from non-tumor, prior to macro-dissection of each sample.

### 2.2. Clinical Data Collection

Clinical data were collected for all specimens and included primary oropharyngeal subsite, sex, age at diagnosis, race/ethnicity, tumor grade, smoking history, and HPV/p16 status. Tumor, Node, and Metastasis (TNM) staging and overall stage was recorded using the standard *AJCC*, *Eighth Edition*. Vital status (living or deceased) and tumor status was recorded for the last known clinic visit/contact. Patients who were reported as lifelong non-smokers were termed “never smokers,” and all other patients were considered smokers (current/former). 

### 2.3. Sample Processing

FFPE samples were submitted for analysis using the path HTG EdgeSeq panel and macro-dissected to include the area of interest (HTG Molecular Diagnostics Inc., Tucson, AZ, USA). The area of each sample was measured and HTG Lysis Buffer added, to obtain a per-well concentration of 6 mm^2^/35 µL. To improve sample lysis, Proteinase K was added at 1/20th Lysis Buffer volume and samples incubated at 50 °C for 180 min. Thirty-five µL of each sample was added to a single well of a 96-well plate. Human Universal RNA was also added to three wells at 25 ng, to serve as a process control. 

Samples were run on an HTG EdgeSeq Processor, using the HTG EdgeSeq PATH gene panel. Following the processor step, samples were individually barcoded (using a 19-cycle PCR reaction to add adapters and molecular barcodes). Barcoded samples were individually purified using AMPure XP beads and quantitated using a KAPA Library Quantification kit. The library was sequenced on an Illumina MiSeq using a V3 150-cycle kit with two indexes reads. PhiX was spiked into the library at 5%; this spike-in control is standard for Illumina sequencing libraries. After assessing the adequacy of sample/sequencing and quality control, 32 out of 37 samples were included in the final analysis. In total, 3 samples which had failed the sequencing quality control and 2 genetic outliers were excluded from the statistical analysis. 

### 2.4. Data Analysis

Data are returned from the sequencer in the form of de-multiplexed FASTQ files, with one file per original well of the assay. The HTG EdgeSeq Parser was used to align the FASTQ files to the probe list to collate the data. Data from the company were provided as raw, quality-control (QC) raw, counts per million (CPM), and median normalized and fold-change data. P-values were calculated for each probe after adjustment, using the Benjamini and Hochberg (1995) method for controlling discovery rate. Normalized counts of samples were used for single sample geneset enrichment with a predefined human immune cell geneset through the Genepattern ssGSEA portal as previously described [21,22]. Immune cell populations of the four groups (HPV+R-, HPV+R+, HPV-R- and HPV-R+) were then plotted with pheatmap [23]. The immune-cell-population-enriched score of patient groups were calculated using GSEA [24]. Briefly, the following gene sets were used to assign cell lineages: Myeloid/Monocyte Progenitor: F2RL1,CD34, IFIT1,TPSAB1, IL4, CD28, BID, CXCL14, TGFB2, IL3, FCER1A, MPO, NRP1, LIF, MAPKAPK2, CD33, KIT, TWIST1, CTSG, RUNX1, TNFSF11, CSF2R, CCL19, EGFR, IGLL1, IL5RA, IL1B, and MS4A2. Monocyte: F13A1, S100P, LMO2, FCGR1A, PTGS2, CD163, FCGR2A, TIMP1, ALDH1A1, TLR2, HLA-DRA, CD14, ICAN1, CD4, CTSSB, ENTPD1, CD86, CKAP4, S100A6, S100A8, CDK1A, GSTP1, ITGAX. CD9, THBD, SERPINA1, and CDA. Megakaryocyte: F13A1, S100P. FCGR2A, TTMP1. CD8A. SLC2A3, S100A8,ITGB3, SNCA, CD9, MPL, MTTF, SELP, GZMB, PRF1, GYPA, LTF, and ADORA2A. Granulocyte progenitor cell (Neutrophilic-metamyelocyte): F13A1, ITGAM, FCGR1A, TLR2,S100P, CD163, ALDH1A1, FCGR2A, PTGS2, CD14, CTSB, TOP2A, CKAP4, CD4, ITGAX, S100A8, CD86, CD8A, CD9, ENTPD1, CDKN1A, DPYD, MPL, LTF, MS4A1, CDA, and SERPINA1. Granulocyte-monocyte progenitor: F13A1, SLAMF7, LMO2, ITGAM, TPSAB1, TPSAR1, PTGS2, CD163, FCGR2A, CD34, TDH1, ANXA1, TLR2, HLA-DRA, CD63, STMN1, C100A6, C100A8, FLT3, VTM, ITGAX, SNCA, TRF8, MPL, MTTF, SELP, HLA-DQA1, and MPO.

For TCGA analysis: Gene expression of head-and-neck patients were downloaded from the TCGA head and neck cancer RNAseq dataset (https://portal.gdc.cancer.gov/projects/TCGA-HNSC (accessed on 21 October 2019) and run through the same analysis pipeline for enrichment scores as described for the patient samples. Tumor HPV status was assessed for the TCGA cohort, as previously published [25].

## 3. Results

### 3.1. Demographics and Clinical Parameters

A total of 37 patient samples (14 = HPV+R+, 12 = HPV+R-, 5 = HPV-R+, and 6 = HPV-R-) were retrospectively collected and analyzed for clinical and demographic variables, including HPV status, race, gender, smoking history, subsite, TNM staging, overall stage, treatment, disease-free and overall survival. A total of 81% of the cohort was male and 26 (70%) had HPV+ cancer. The majority were Caucasian (84%). Most patients were also current or former smokers (62%) vs. never-smokers (38%). The primary subsite was the tonsil (n = 27), followed by the tongue base (n = 10). A total of 73% (n = 27) underwent chemoradiotherapy as their definitive treatment, while the remainder underwent primary radiotherapy (Table 1). The HPV+ group (n = 26) was separated into two groups based on recurrence, and clinical factors were examined using logistic regression. Gender, smoking status, subsite, N status and treatment were not associated with recurrence (*p* > 0.05). While some of the clinical characteristics are variable, including treatment, advanced T stage was the only factor that was associated with recurrence in our cohort (95% CI, 0–0.9, *p* = 0.035) (Table 2). In the HPV+R+ cohort, the disease-free survival was 10.8 months, and all patients died of the disease, with an overall survival of 27.5 months. A total of 25% of patients had local recurrence, 25% had loco-regional recurrence and distant metastasis, and 50% had distant metastasis. Distant metastatic sites were lung (most common) followed by bone and brain.

### 3.2. GeneExpression

#### 3.2.1. Suppressive Myeloid Cell Signatures Are Enriched in Aggressive OPSCC

**Figure 1:**
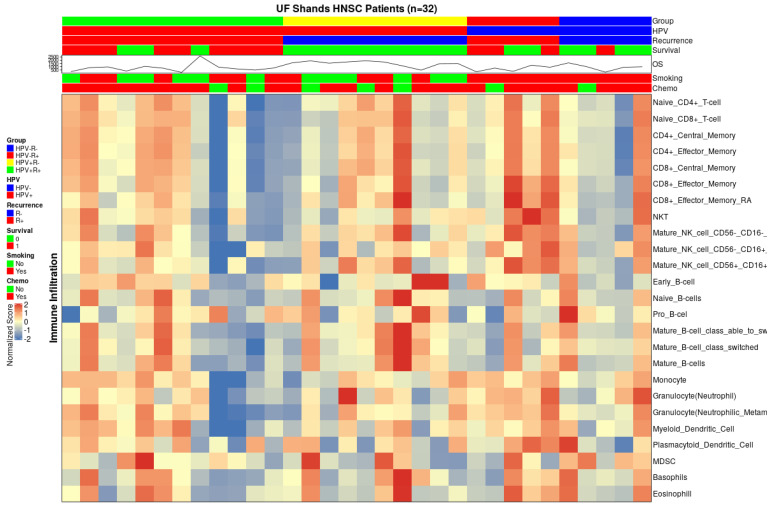
Enrichment of immune cells in oropharynx cancer patients based on HPV and recurrence status demonstrates increased suppressive monocyte/macrophage cell signatures. The heatmap demonstrates overall higher expression of myeloid cell expression pathways in HPV+R+ patients when compared to the other cohorts, specifically in the monocytic and T cell pathways. Suppressive monocyte and myeloid cell progenitor enrichment resulted in significant differences.

**Figure 2:**
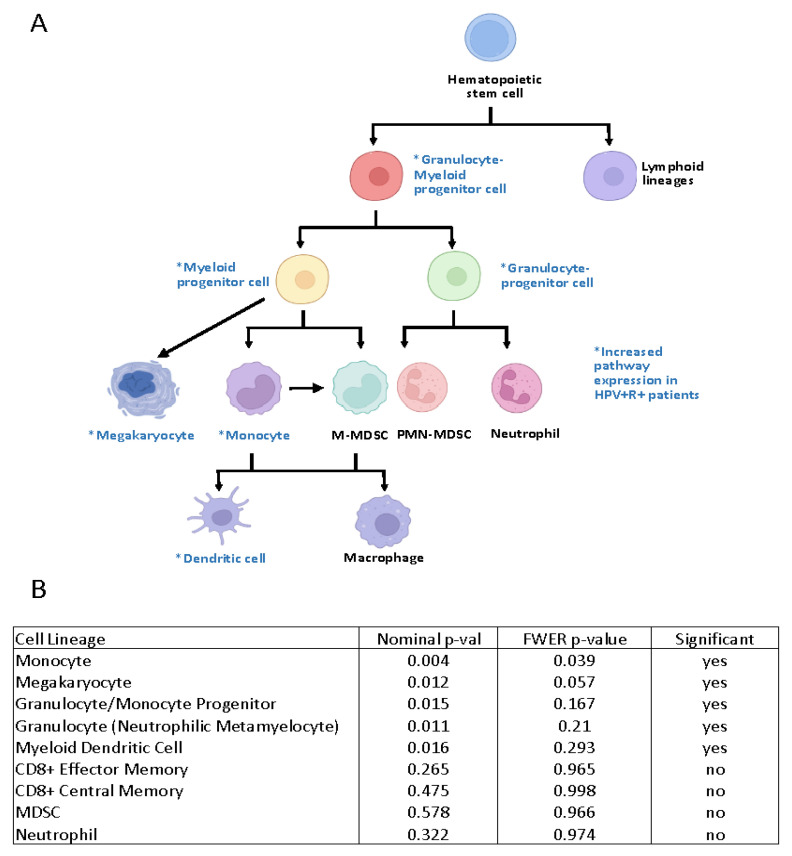
Summary of enriched granulocyte-myeloid cell lineages in HPV+R+ patients vs. HPV+R-. (**A**). GSEA analysis revealed enrichment of granulocyte-myeloid pathways in HPV+R+ patients, including myeloid and granulocytic progenitor cells, which give rise to suppressive monocytes/macrophages and MDSCs. (**B**). List of cell lineages and significance levels for pathways evaluated. *=increased expression of pathway in HPV+R+ patients.

A total of 32 patients had adequate sequencing results and were included in the genetic analysis. The fold-change expression of phenotypic markers for various immune cell populations of the four clinical groups of patients (HPV+R+; HPV+R-; HPV-R+; and HPV-R-) were plotted, using pheatmap. Overall, there was clustering of increased gene expression in both the myeloid cell and T cell populations in the HPV+R+ patients (Figure 1). However, GSEA analysis of HPV+R+ vs HPV+R- patients resulted in significant enrichment of granulocyte-myeloid pathways, monocyte and megakaryocyte pathways. T cell pathways were not significantly altered in HPV+R+ patients (Figure 2). 

**Figure 3:**
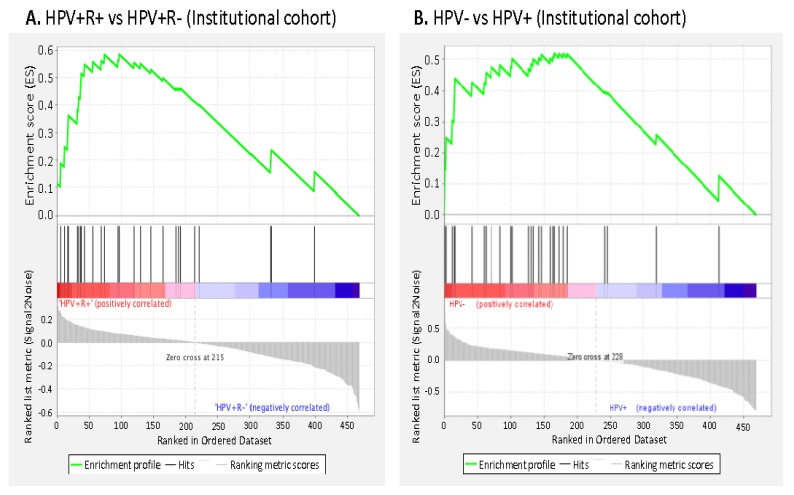
Suppressive Monocytes/macrophages are significantly enriched in HPV- patients and HPV+R+ patients. Using GSEA pathway analysis, in the institutional cohort: (**A**). HPV+R+ patients showed a significant correlation, with an enriched suppressive monocytic pathway when compared to HPV+R- (nominal *p*-value = 0.004, FWER *p*-value = 0.039). (**B**). HPV- patients, when compared to HPV+ patients, demonstrated a similar enrichment in suppressive monocyte cell pathways (nominal *p* value < 0.001, FWER *p*-value = 0.014).

Suppressive monocytes were significantly enriched in HPV+R+ patients (*p* = 0.004, family-wise error rate (FWER), *p*-value = 0.039) when compared to HPV+R- patients (Figure 3A). Regardless of recurrence status, GSEA analysis demonstrated a significant enrichment of suppressive monocytes in the HPV-negative patients (all) when compared to HPV+ patients (all) in the institutional patient cohort (nominal *p*-value < 0.001 (FWER), *p*-value = 0.014) (Figure 3B). 

#### 3.2.2. Gene Expression Profiling Demonstrated Increases in M2-Like Macrophage-Associated Genes in Patients with Recurrent HPV+ Cancer

**Figure 4:**
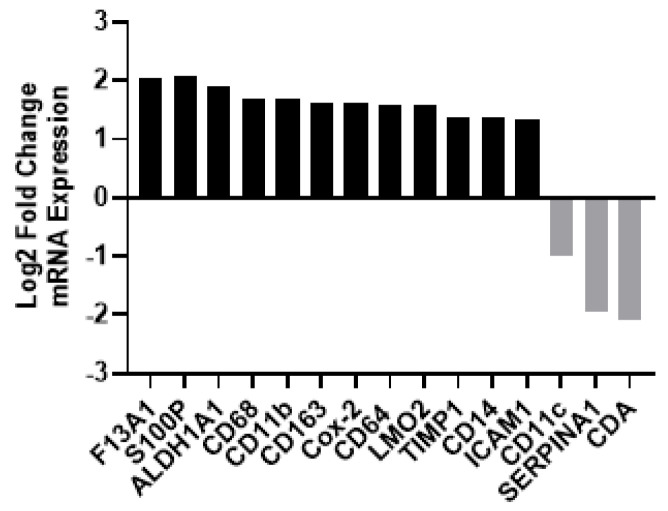
Fold-Change Gene Expression of Suppressive Monocyte Associated Genes in HPV+R+ versus HPV+R- patients. HPV+R+ patients demonstrated a higher fold-change expression for the genes associated with poor differentiation of monocytes: (F13A1, ALDH1A1); M2 polarization phenotype (PTGS2, CD68, CD163); and decreased expression of M1 macrophage-associated genes (CD11c and CDA).

Suppressive monocytes/macrophages were defined using GSEA analysis, for pre-determined gene sets, and included the following genes: F13A1, S100P, ALDH1A1, CD68, CD11b, CD163, Cox-1, CD64, LMO2, TIMP1, CD14, IACM1, CD11c, SERPINA1, and CDA. We examined individual gene expression data in the monocyte/macrophage pathway in the HPV+ R+ group when compared to the HPV+R- group, and found increased fold change in several genes associated with poor differentiation of monocytes/macrophages. These genes include the monocyte-associated gene F13A1, ALDH1A1, and the suppressive (M2) macrophage polarization markers, Cox-2 (PTGS2) [26], and CD163 [27]. Several other markers were increased, including the macrophage/monocyte markers CD11b, TIMP1, and CD64. On the other hand, the CD11c and CDA genes (associated with the more favorable M1 macrophage subtype) [28] had decreased expression in HPV+R+ patients (Figure 4). In addition, the HPV+R+ groups had non-significant decreased expression of genes associated with somatic recombination, leukocyte adhesion molecules and cell–cell signaling (Supplementary Material Figure S1).

#### 3.2.3. Increased Suppressive Monocyte/Macrophage Cell Expression Is Present in HPV- Negative vs. HPV Positive Patients in the TCGA Cohort

**Figure 5:**
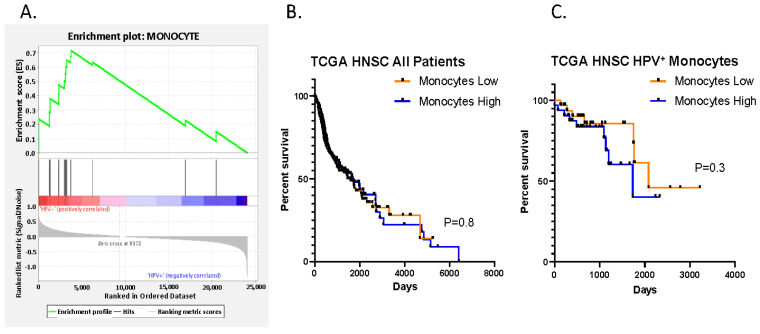
Increased expression of monocytic cell markers is present in HPV-negative vs. HPV-positive patients in the TCGA cohort. (**A**). GSEA analysis revealed a positive correlation of monocytic enrichment in the HPV- patients when compared to HPV+ patients in the TCGA cohort (*p* = 0.0027, FDR q-value = 0.0059, FWER *p*-value = 0.014). (**B**,**C**). There was no correlation observed with the survival among all head-and-neck-cancer patients analyzed and monocyte status (high vs. low); *p* = 0.8 with all patients and within the HPV+ patient group (*p* = 0.3).

We performed GSEA pathway analysis on expression data available from the head-and-neck-cancer TCGA database comprised of 499 patients (all head-and-neck subsites) which included 64 patients recorded as HPV+. HPV-negative patients had increased suppressive monocyte/macrophage pathway enrichment (nominal *p*-value = 0.002, and FWER *p*-value = 0.014) when compared to HPV+ patients. After grouping the patients into monocyte “hi” vs “low”, based on median expression, there was no difference in survival according to the presence of suppressive monocyte/macrophage cells (Figure 5A–C). 

**Figure 6:**
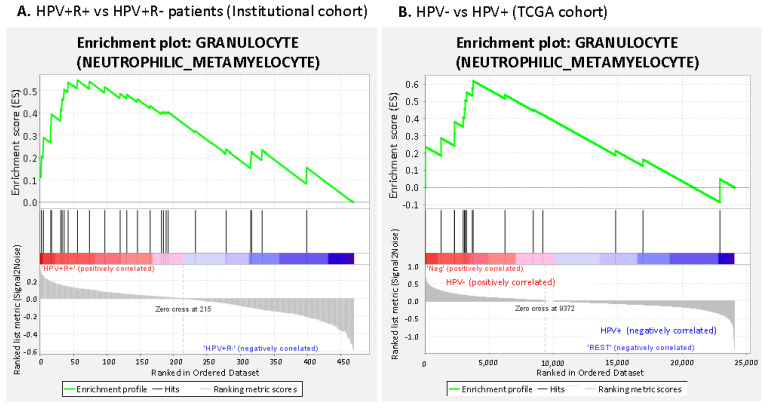
Enrichment of myeloid progenitor cell expression profiles is demonstrated in aggressive OPSCC tumors. (**A**). GSEA analysis revealed enrichment of myeloid progenitor cell expression (neutrophilic metamyelocyte) in the HPV+R+ patients when compared to HPV+R- patients (*p* = 0.0, FDR q-value 0.004, FWER *p*-value = 0.03). (**B**). In the TCGA cohort, HPV- patients were correlated with enriched myeloid progenitor cell signatures when compared to HPV+ patients (*p* = 0.0, FDR q-value 0.0052).

Granulocytes (specifically neutrophilic metamyelocytes), were also enriched in HPV+R+ compared to HPV+R- patients (Figure 6A). Additionally, in the TCGA dataset a similar pattern was found, in which this cell lineage was enriched in the more aggressive HPV- tumors when compared to HPV+ tumors (Figure 6B). 

## 4. Discussion

The vast majority of HPV-positive OPSCC patients are cured with standard therapy [3]. When patients do have recurrence, the disease is often fatal. It is important to investigate the immune landscape for HPV+ patients who develop recurrence and metastasis, in order to identify factors that may contribute to worse clinical outcomes, so that we may develop tailored therapies for this population. In the present study, we identified immunologic factors that are associated with recurrence in a cohort of OPSCC patients treated at a single institution and using the TCGA database. Our results show a significant increased gene enrichment in the suppressive monocytic/macrophage cell pathway and granulocyte cell pathway in patients with HPV+ recurrent disease and HPV-negative disease (in the institutional cohort), and in HPV-negative patients when compared to HPV+ patients (in the TCGA cohort). Since patients with recurrent HPV+ cancer have a more aggressive disease that clinically resembles more closely the HPV-negative patients, the finding of upregulation of the suppressive immune cell pathways in both of these cohorts may be an important factor associated with overall worse clinical outcomes, and could potentially serve as a prognostic biomarker. 

The balance of anti-tumoral myeloid cells (e.g., M1 macrophages) and pro-tumorigenic immunosuppressive cells (e.g., MDSCs and M2 macrophages) can alter the immune microenvironment, and may play a key role in treatment response. While HNSCCs overall are considered more immune-cell-rich tumors when compared to other solid tumors (especially in HPV+ patients), HNSCCs are also rich in immunoregulatory cells that can dampen anti-tumor immune responses [29]. T-cell infiltration (especially CD8+ lymphocytes) has been associated with a favorable prognosis in HNSCC and other cancer types [7,8,9,10]. Additionally, studies have demonstrated that monocyte-to-lymphocyte ratio in the blood correlates with worse prognosis [30,31,32,33]. In our study, there were no significant differences between expression of T lymphocyte pathways in HPV+R+ groups and HPV+R- groups. In fact, there was a non-significant increase in the overall inflammatory signature in HPV+R+ patients. HPV+R+ patients may represent an immune-cell rich, but immune-dysfunctional, and overall immunosuppressive tumor micro-environment. 

Tumor-associated macrophages (TAMS) are a major component of the tumor immune microenvironment, but there is still much to learn about the role of macrophages in tumor progression. CD68 and CD163 are the biomarkers most frequently used to determine TAMS in tissue specimens. CD68 is a general marker for TAMS, while CD163 is used to detect M2 polarization of monocytes/macrophages, and CD163 is only expressed on cells from the monocytic lineage [34]. In a recent meta-analyses of TAMs in HNSCC, high stromal expression of CD163+ TAMS correlated with poor overall survival and progression-free survival, based on immunohistochemistry [35]. Additionally, a higher density of both the total and M2-like subtype of TAMs in the tumor microenvironment was associated with advanced T stage, increased rates of nodal positivity, the presence of vascular invasion, and the presence of lymphatic invasion [36]. In our institutional cohort, we observed a suppressive monocyte/macrophage signature in HPV+R+ patients as well as in HPV-negative (all), based on a suppressive immune signature characterized by the increased mRNA expression of CD163. The only significantly different clinical parameter between the HPV+R+ and HPV+R- group was that the HPV+R+ group was more likely to have advanced T stage, a finding which may be strongly associated with an increased TAM signature. When we examined clinical outcomes in the TCGA cohort, we did not find a statistically significant association between “hi” suppressive monocytic infiltration and overall survival, implying that prognostication of worse outcomes is likely multifactorial and potentially dependent on immune cell ratios. 

Focusing on monocytes/TAMs as a therapeutic target may be effective in more aggressive diseases such as OPSCC patients with HPV+ disease with recurrence and HPV-negative HNSCC patients. The monoclonal antibodies anti-programmed death protein-1 (anti–PD-1) nivolumab and pembrolizumab are the first immune checkpoint inhibitors (ICIs) approved for treatment of recurrent/metastatic (R/M) HNSCC. Clinical experience in HNSCC demonstrates ~20% ORR for anti-PD-1 inhibition in the R/M setting [37,38]. PD-1 blockade has been effective in the treatment of aggressive HNSCCs, but the majority of patients still progress. Since TAMs can be abundant in HNSCCs, strategies aimed at blocking TAMs, in addition to a PD-1 checkpoint blockade, may be critical. Some strategies under investigation are aimed at reprogramming or repolarizing TAMs from the M2 pro-tumoral phenotype toward M1 anti-tumoral phenotype. Some active clinical trials targeting macrophages have HNSCC patient cohorts (e.g., NCT02216409, agent Hu5F9-G4 targeting CD47, and NCT02829723 agent BLZ945 targeting CSF1R). 

We recognize certain limitations in our study, including small sample size and differences in patient clinical features, including primary treatment. Additionally, the nature of gene expression may not correlate with end protein expression, due to post-transcriptional or posttranslational modifications, and the results of monocyte/macrophage expression have not been confirmed at the protein level. While we are able to appreciate genetic signatures that correlate with suppressive monocytes/macrophages and myeloid-granulocyte signatures, due to the limitations of the genetic panel used in this study we were unable to further sub-classify and identify specific myeloid and granulocyte cell subsets, due to the gene panel used (i.e., M-MDSC. PMN-MDSCS, M1/M2 macrophages). 

## 5. Conclusions

In conclusion, this study demonstrates a suppressive myeloid/monocyte and granulocyte gene expression enrichment in patients with aggressive oropharynx cancer (recurrent HPV+ and HPV-negative). Further investigation into targeting these cells in combination with T-cell activating therapies may provide a beneficial therapeutic combination for these hard-to-treat patients. 

## Figures and Tables

**Table 1:** Patient Demographic and Clinical Characteristics.

	Patient Characteristics		
		N=37	% (Total)
**Gender**	Male	30	81
	Female	7	19
**HPV status**	Negative	11 (6 with recurrence)	30
	Positive	26 (14 with recurrence)	70
Race	White	31	84
	African American	4	11
	Other	2	5
**Smoking history**	Never	14	38
	Current or former	23	62
**Subsite**	Tongue Base	10	27
	Tonsil	27	73
**N Status**	Negative	9	24
	Positive	28	76
**Overall Stage**	1	11	30
	2	6	16
	3	13	35
	4	7	19
**Treatment**	Chemoradiotherapy	27	73
	Radiation alone	10	27

**Table 2:** Logistical Regression (recurrence vs. no recurrence for the HPV+ OPSCC patients).

Risk Factor			No Recurrence N = 12	Recurrence N = 14	OR	Lower CI	Upper CI	*p*-Value
**Gender**	Male		10	14	5.9	0.1	263.8	0.362
	Female		2	0				
**Smoking Status**	Never		8	6	2.4	0.5	11.5	0.278
	Former/current		4	8				
**Subsite**	Tonsil		11	9	0.2	0	1.6	0.124
	Tongue		1	5				
**T Stage**	1, 2		9	3	0.2	0	0.9	**0.035**
	3, 4		3	11				
**N Status**	Negative		2	2	0.2	0.1	8.4	1
	Positive		10	12				
**Treatment**	CRT		7	12	3	0.5	18.7	0.245
	RT		5	2				

## Data Availability

The TCGA head-and-neck-cancer database was accessed.

## Supplementary Materials

The following supporting information can be downloaded at https://www.mdpi.com/article/10.3390/cancers15082346/s1, Figure S1: Gene expression profiles of immune and cell-cycle pathways in HPV+R+ vs HPV+ R- patients.
