# Supplementary figures and images for: Association of Suppressive Myeloid Cell Enrichment with Aggressive Oropharynx Squamous Cell Carcinoma
Source: Cancers (Basel). 2023 Apr 18;15(8):2346. doi: 10.3390/cancers15082346 (PMC10136648; doi:10.3390/cancers15082346)

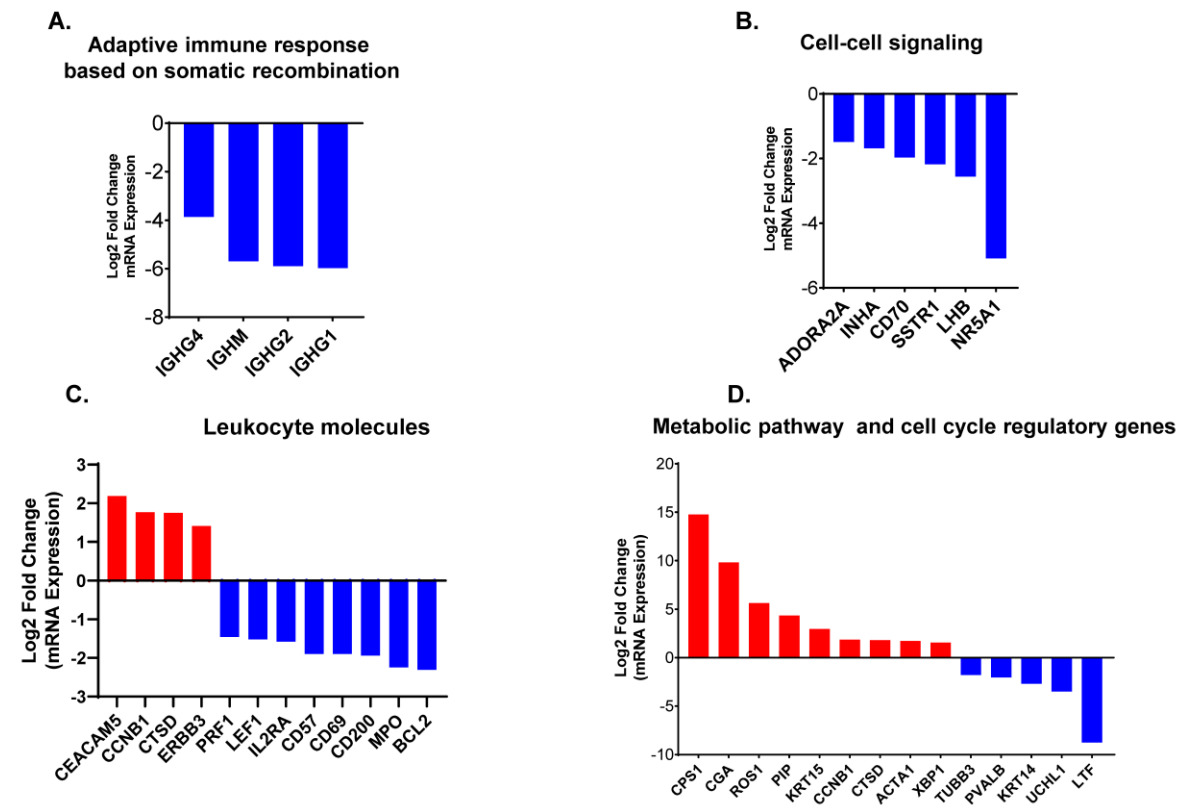

Figure S1: Gene expression profiles of immune and cell-cycle pathways in HPV+R+ vs HPV+ R- patients.

Supplement: Supplementary file 1 [file cancers-15-02346-s001.zip › cancers-2235161-supplementary.pdf]
